# A nomogram for predicting severe myelosuppression in small cell lung cancer patients following the first-line chemotherapy

**DOI:** 10.1038/s41598-023-42725-7

**Published:** 2023-10-14

**Authors:** Yaoyuan Li, Yanju Bao, Honggang Zheng, Yinggang Qin, Baojin Hua

**Affiliations:** Department of Oncology, Guang’anmen Hospital, China Academy of Chinese Medical Sciences, Beixiange 5, Xicheng District, Beijing, 100053 China

**Keywords:** Cancer, Diseases

## Abstract

This study aimed at establishing and validating a nomogram to predict the probability of severe myelosuppression in small cell lung cancer (SCLC) patients following the first-line chemotherapy. A total of 179 SCLC cases were screened as the training group and another 124 patients were used for the validation group. Predictors were determined by the smallest Akaike’s information criterion (AIC) in multivariate logistic regression analysis, leading to a new nomogram. The nomogram was validated in both training and validation groups and the predicting value was evaluated by area under the receiver operating characteristics (ROC) curve (AUC), calibration curve, and decision curve analysis (DCA). Age and tumor staging were extracted as predictors to establish a nomogram, which displayed the AUC values as 0.725 and 0.727 in the training and validation groups, respectively. This nomogram exhibited acceptable calibration curves in the two groups and its prediction added more net benefits than the treat-all scheme and treat-none scheme if the range of threshold probability in the DCA was between 15 and 60% in the training and validation groups. Therefore, the nomogram objectively and accurately predict the occurrence of severe myelosuppression in SCLC patients following the first-line chemotherapy.

## Introduction

Small cell lung cancer (SCLC) accounts for approximately 15% of lung cancer cases^1^ and is characterized by rapid growth and early distant metastasis^2,3^. Furthermore, many SCLC patients are diagnosed usually at advanced stages (approximately, 70% of the SCLC patients are at stage IV) and the mortality is about 95%^1,4^. While targeted therapies and immunotherapies have successfully improved the survival of non-SCLC patients, there has been no significant therapeutic breakthrough for SCLC patients in the last three decades^2,5^.

Currently, patients with SCLC depend on chemotherapies, such as the first line etoposide and cisplatin (EP) or carboplatin (EC) for patients at limited stage (LS-SCLC) and extensive stage (ES-SCLC)^6–8^. Recent studies have shown that the combination of chemotherapies with immunotherapies, such as atezolizumab and durvalumab, can prolong the survival of patients with ES-SCLC, compared to chemotherapy alone^6,9,10^. However, these therapeutic strategies are just approved by the US Food and Drug Administration^11^ and have not yet become routine clinical strategies in many other regions. Moreover, chemotherapy is still indispensable in combination therapy. It is well known that these chemotherapies had severe adverse effects, such as severe myelosuppression. The severe myelosuppression (≥ 3 degrees) is characterized by hemoglobin (HB) < 80 g/L or white blood cells (WBC) < 2 × 10^9^/L or neutrophil < 0.5 × 10^9^/L or platelets (PLT) < 50 × 10^9^/L, according to the criteria of acute and subacute anticancer drug toxicity of the World Health Organization (WHO). Patients with severe myelosuppression display symptoms of severe bone marrow suppression and require timely intervention to avoid major harm. For example, neutropenia and thrombocytopenia are common in patients with severe myelosuppression and complicated with infection, which can be dangerous and even lead to death. More importantly, in some patients, severe myelosuppression leads to early discontinuation, which can reduce clinical benefits. Given the necessity of these chemotherapy interventions for SCLC patients and the harmfulness of chemotherapy-induced severe myelosuppression, it is urgent to determine the factors that can predict severe myelosuppression in SCLC patients following the first line chemotherapies. Unfortunately, there are no reliable biomarkers and methods for evaluating the occurrence of severe myelosuppression in SCLC patients.

Nomogram is a graphical presentation of the relationship among multiple variables by scales from statistical models and widely used for prognosis in oncology and medicine^12^. Because the nomogram can generate a specific probability of a clinical event for a special person, it is valuable for personalized medicine^5,12^. However, there is not a nomogram available for predicting the occurrence of severe myelosuppression in SCLC patients following the first-line chemotherapy. This study aimed at developing and validating a nomogram to predict the probability of occurrence of severe myelosuppression in SCLC patients following the first-line chemotherapy (EP or EC regimen), and to provide individualized treatment decisions.

## Methods

### Patients and study design

The training group included 179 cases who were pathologically diagnosed as inoperable SCLC and received EP or EC standard chemotherapy in the oncology department of our hospital from 2013 to 2018. The inclusion criteria included complete medical records, no history of chemoradiotherapy for the disease, HB ≥ 95 g/L and WBC ≥ 3 × 10^9^/L and PLT ≥ 80 × 10^9^/L, and karnofsky performance status (KPS) ≥ 60 points. The exclusion criterion was acceptance of other chemotherapeutic regimens (besides EP/EC).

Another group of 124 cases was screened from January 2019 to April 2021 using the same inclusion and exclusion criteria for validation of the nomogram.

The protocol was performed in accordance with the guidelines outlined in the Declaration of Helsinki and was approved by the Ethics Committee of Guang’anmen Hospital, China Academy of Chinese Medical Sciences. Since the study was a retrospective study, most of the study subjects have died or lost contacts, and all statistics were anonymous, so the Ethics Committee of Guang'anmen Hospital, China Academy of Chinese Medical Sciences agreed to waive the need for informed consent.

### Outcome definition

According to conventional chemotherapy procedures, peripheral blood samples were collected before and within 21 days after chemotherapy to assess bone marrow suppression caused by chemotherapy. Individuals with a grade 3 or 4 of HB reduction (< 80 g/L), leukopenia (< 2 × 10^9^/L), neutropenia (< 0.5 × 10^9^/L), or thrombocytopenia (< 50 × 10^9^/L) at any time within 21 days after chemotherapy were considered as severe myelosuppression patients.

### Demographic and clinical measures for prediction and definition

Potential demographic and clinical measures for prediction included categorical variables such as gender, age, current smoking status, body surface area (BSA), body mass index (BMI), tumor staging, malignant pleural effusion, liver metastasis, bone metastasis, HB, serum neuron-specific enolase (NSE) levels, number of chemotherapy and chemotherapy regimens, as well as continuous variables such as serum albumin (ALB) levels and KPS. These measures were stratified in Table 1. BMI ≥ 23 kg/m^2^ was defined as overweight, according to Asian criteria^13^. SCLC was staged according to the Veterans Administration Lung Study Group (VALG) staging system. SCLC at limited stage (LS) was determined when the lesion was restricted in one hemithorax and one radiation field, but not with ipsilateral pleural effusion. SCLC at extensive stage (ES) meant that the lesion extended > one hemithorax. The normal values were HB ≥ 130 g/L (male) or HB ≥ 115 g/L (female), ALB ≥ 40 g/L and NES < 16.3 ng/mL in our hospital. The number of chemotherapy cycles was divided into three categories: 1st or 2nd; 3rd or 4th; 5th and above. Patients with the EP or EC chemotherapy were treated with 60–100 mg/m^2^ of etoposide on days 1–5 or 1–3 and 20–30 mg/m^2^ of cisplatin on days 1–3 (EP) or 5–6 mg/ml/min AUC of carboplatin on day 1 (EC) every three weeks, respectively.

**Table 1:** Clinical characteristics of patients in the training and validation groups.

Characteristics	Training group			Validation group		
	NSM (n = 124)	SM (n = 55)	P-value	NSM (n = 88)	SM (n = 36)	P-value
Age (y)			< 0.01			0.15
< 60	46 (37.1%)	6 (10.9%)		15 (17%)	3 (8%)	
60–74	55 (44.4%)	33 (60.0%)		57 (65%)	21 (58%)	
≥ 75	23 (18.5%)	16 (29.1%)		16 (18%)	12 (33%)	
Gender			0.60			0.39
Male	87 (70.2%)	36 (65.5%)		64 (73%)	23 (64%)	
Female	37 (29.8%)	19 (34.5%)		24 (27%)	13 (36%)	
Current smoking			0.87			0.15
No	55 (44.4%)	23 (41.8%)		38 (43%)	10 (28%)	
Yes	69 (55.6%)	32 (58.2%)		50 (57%)	26 (72%)	
BSA, median (IQR) (m^2^)	1.8 (1.6, 1.8)	1.7 (1.6, 1.8)	0.47	1.8 (1.6, 1.8)	1.7 (1.5, 1.8)	0.23
KPS, median (IQR)	90.0 (80.0, 90.0)	80.0 (80.0, 90.0)	0.04	90.0 (80.0, 90.0)	80.0 (80.0, 90.0)	0.03
BMI			0.22			0.07
Not overweight	33 (26.6%)	20 (36.4%)		30 (34%)	19 (53%)	
Overweight	91 (73.4%)	35 (63.6%)		58 (66%)	17 (47%)	
Tumor staging			< 0.01			< 0.01
LS	38 (30.6%)	6 (10.9%)		30 (34%)	2 (6%)	
ES	86 (69.4%)	49 (89.1%)		58 (66%)	34 (94%)	
Pleural effusion			0.06			0.30
Not	88 (71.0%)	31 (56.4%)		59 (67%)	20 (56%)	
Yes	36 (29.0%)	24 (43.6%)		29 (33%)	16 (44%)	
Bone metastasis			0.47			0.05
Not	91 (73.4%)	37 (67.3%)		66 (75%)	20 (56%)	
Yes	33 (26.6%)	18 (32.7%)		22 (25%)	16 (44%)	
Liver metastasis			0.06			0.22
Not	98 (79.0%)	36 (65.5%)		73 (83%)	26 (72%)	
Yes	26 (21.0%)	19 (34.5%)		15 (17%)	10 (28%)	
HB			0.24			0.08
Normal	80 (64.5%)	30 (54.5%)		45 (51%)	12 (33%)	
Abnormal	44 (35.5%)	25 (45.5%)		43 (49%)	24 (67%)	
ALB			1.00			0.69
Normal	70 (56.5%)	31 (56.4%)		33 (38%)	15 (42%)	
Abnormal	54 (43.5%)	24 (43.6%)		55 (63%)	21 (58%)	
NSE			0.62			0.83
Normal	68 (54.8%)	33 (60.0%)		61 (69%)	24 (67%)	
Abnormal	56 (45.2%)	22 (40.0%)		27 (31%)	12 (33%)	
No. of chemo			0.80			0.61
1st or 2nd	64 (51.6%)	26 (47.3%)		37 (42%)	14 (39%)	
3rd or 4th	47 (37.9%)	22 (40.0%)		29 (33%)	15 (42%)	
5th and above	13 (10.5%)	7 (12.7%)		22 (25%)	7 (19%)	
Chemo regimen			0.39			0.05
EC	84 (67.7%)	33 (60.0%)		66 (75%)	20 (56%)	
EP	40 (32.3%)	22 (40.0%)		22 (25%)	16 (44%)	

*NSM* non-severe myelosuppression, *SM* severe myelosuppression, *BSA* body surface area, *KPS* karnofsky perform status, *BMI* body mass index, *LS* limited stage, *ES* extensive stage, *HB* hemoglobin, *ALB* albumin, *NSE* neuron specific enolase, *EP* etoposide plus cisplatin, *EC* etoposide plus carboplatin.

### Statistical analysis

Data are expressed as the real value, percent, median and interquartile range (IQR). Continual values were analyzed by the Mann–Whitney *U* test while those categorical values were analyzed by Fisher’s exact test. The potential association between variates was analyzed by univariate analysis and predictors were further determined by multivariate logistic regression analysis, leading to a new nomogram. Forward, backward and stepwise selections were applied by using the likelihood ratio test with Akaike’s information criterion (AIC) as the stopping rule^14^.

Individual predictions were evaluated by the area under the receiver operating characteristic (ROC) curve (AUC) with a range of 0 to 1 to determine the degrees of discrimination. The relationship between the observed outcomes and the predicted probabilities was evaluated by the calibration curves using the unreliability U test. A calibration curve can be the best fit when its intercept α = 0 and slope β = 1. The clinical usefulness of the nomogram was examined by decision curve analysis (DCA)^15^.

All statistical analyses were performed using R version 3.6.1 (http://www.r-project.org) and STATA 15.0 for Windows (Stata Corp Texas, USA). The ‘rms’ package in R was used for nomogram and calibration curve. A two-sided P-value of < 0.05 was considered statistically significant.

## Results

### Risk factors for severe myelosuppression in SCLC patients following the first-line chemotherapy

To determine the potential risk factors for severe myelosuppression, we recruited 179 and 124 cases for training and validation, respectively, using the same inclusion and exclusion criteria. The patient demographic and clinical characteristics were shown in Table 1. There were no significant differences in the percentages of patients with severe myelosuppression between the two groups (30.73% vs. 29.03%, P = 0.75).

Univariate analyses indicated that age (≥ 60 years old, P < 0.01), lower KPS (P = 0.04), tumor staging (ES type, P < 0.01), but not other measures, were significantly associated with severe myelosuppression in the training group of SCLC patients following the first-line chemotherapy. In the multivariate regression analyses, we used three selection procedures (forward, backward and stepwise) to determine the smallest AIC-value. Finally, age and tumor staging were extracted as predictors to enter the prediction model (Table 2).

**Table 2:** Predictors for severe myelosuppression in SCLC patients following the first line chemotherapy.

Intercept and variable	Coef.	P-value	Odds ratio (95% CI)
Age	0.9168821	0.001	2.501 (1.486 to 4.211)
Tumor staging	1.5448	0.002	4.687 (1.752 to 12.542)
Intercept	− 5.438697	–	–

*Coef*. the regression coefficient, *CI* confidence interval.

### A nomogram is generated based on the results of multivariate regression analysis

**Figure 1:**
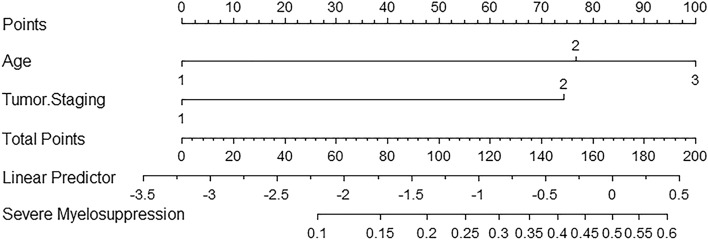
A nomogram for predicting severe myelosuppression in SCLC patients following the first-line chemotherapy. With two predictors associated with severe myelosuppression in SCLC patients following the first-line chemotherapy, a nomogram was established. To use the nomogram, the age and tumor staging classification (Age, 1 =  < 60 y, 2 = 60–74 y, 3 =  ≥ 75 y; Tumor Staging, 1 = LS, 2 = ES) of the individual patient can be located in each variable axis. You can draw a line of each variable classification upward to the “Points” axis to determine its points. Subsequently, in the “Total Points” axis, you can find the total points, from which, you draw a line downward to the “Severe Myelosuppression” axis to obtain the probability of severe myelosuppression.

With the smallest AIC values in multivariate regression analyses, we generated a nomogram with age scales of 1–3 and tumor staging scales of 1–2 (Fig. 1). Besides, there were a point line with scales of 0–100, a total point line with scales of 0–200, and a linear predictor line with scales of − 3.5 to 0.5 as well as a probability line with scales of 0.1 to 0.6. Each variable represented a score corresponding to the point scale, and total points were calculated by summing the scores of each variable. Next, probabilities of severe myelosuppression were estimated by projecting the total points on the probability scale. The application of this nomogram is described in Fig. 1.

### Validation of the nomogram in training and validation groups

**Figure 2:**
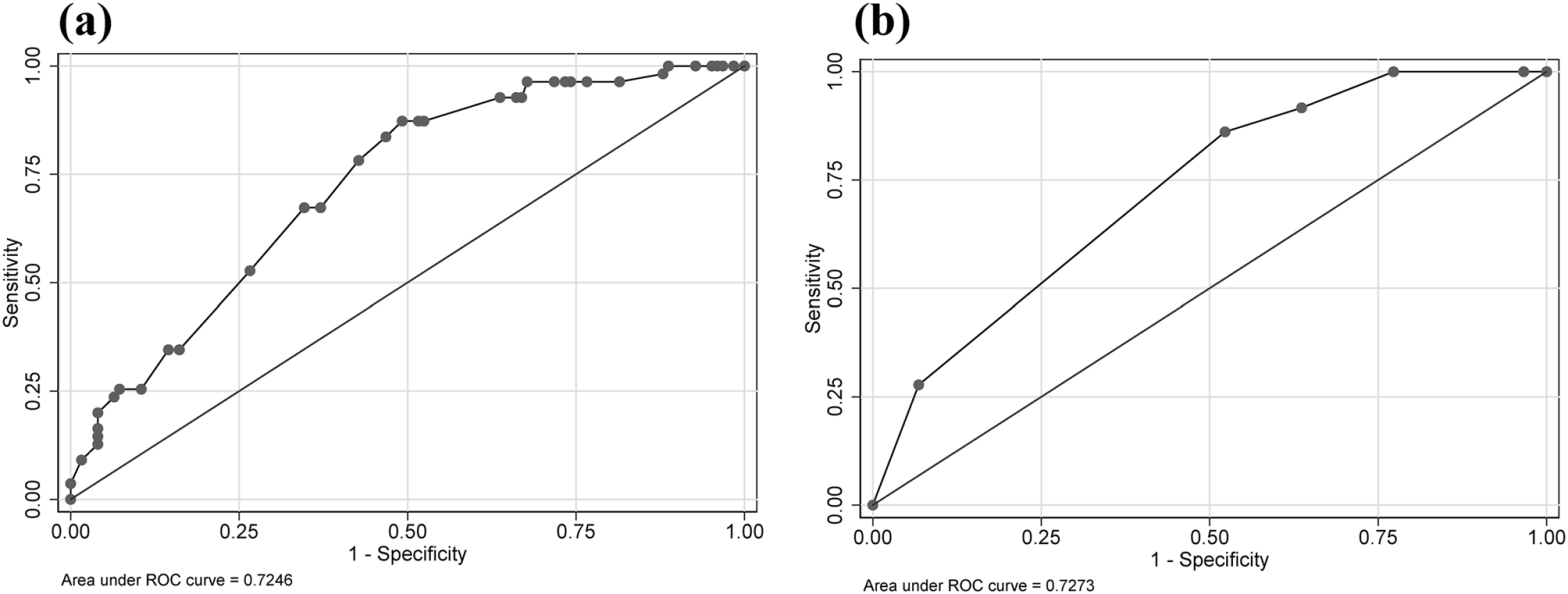
Receiver operating characteristic (ROC) curves in the training and validation groups. (**a**) The ROC curve in the training group with an AUC of 0.725. (**b**) The ROC curve in the validation group with an AUC of 0.727.

**Figure 3:**
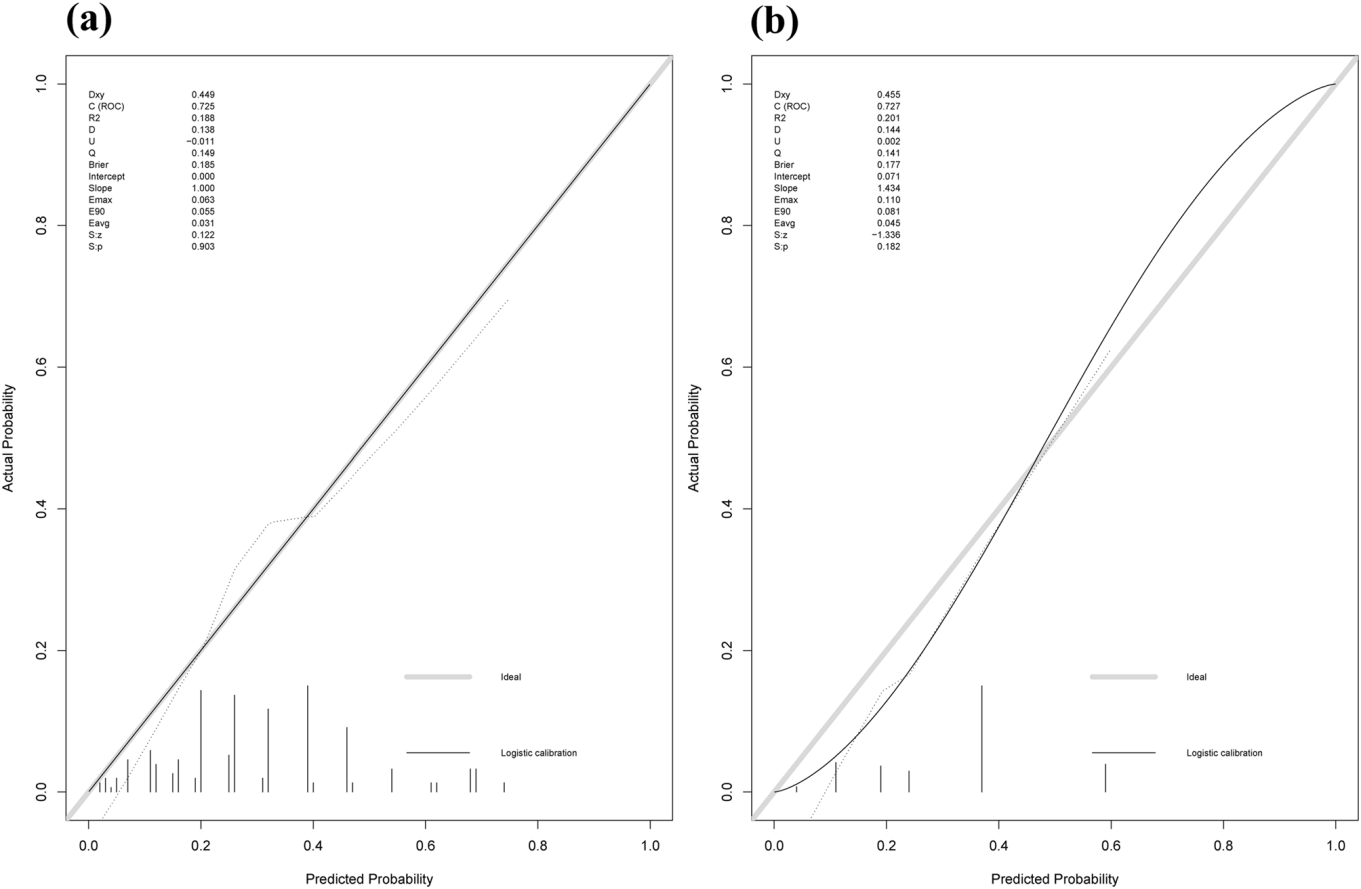
Calibration curves in the training and validation groups. (**a**) The calibration curve in the training group. (**b**) The calibration curve in the validation group. The y-axis represents the actual probability of severe myelosuppression. The x-axis represents the predicted probability of severe myelosuppression. The diagonal thick gray line represents a perfect prediction using an ideal model. The thin solid black line represents the performance of this nomogram, of which, a closer fit to the diagonal thick gray line represents a good prediction.

**Figure 4:**
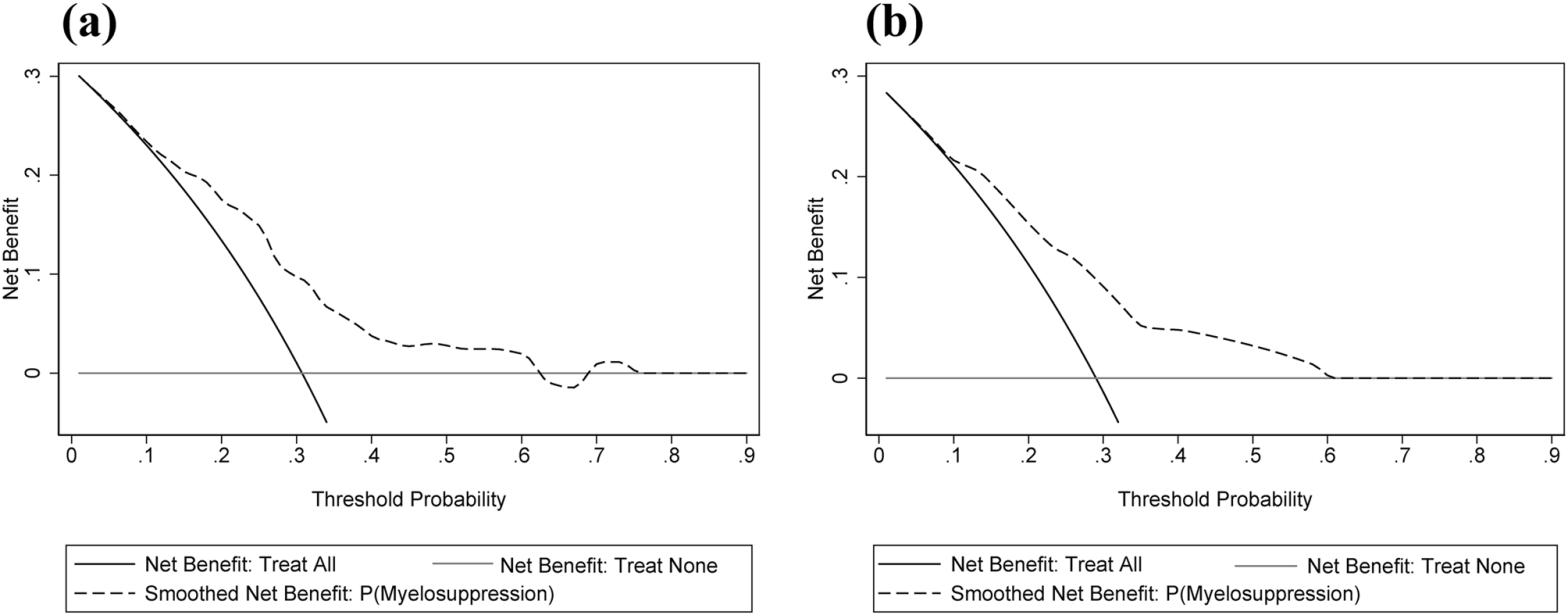
Decision curve analysis of the predictive values of this nomogram in the training and validation groups. (**a**) Decision curve analysis in the training group. (**b**) Decision curve analysis in the validation group. The Y-axis measures net benefit, which was calculated by summing the expected benefits and subtracting the expected harms. The expected benefits are represented by the number of patients, who will have severe myelosuppression and receive clinical intervention to prevent the myelosuppression (true positives). The expected harms are represented by the number of patients without severe myelosuppression, who would be intervened in error (false positives) multiplied by a weighting factor based on the patient’s threshold probability (Pt). The weighting factor captures the patient’s values regarding the risks of under-treatment and over-treatment^26^. The decision curve analysis indicates that a patient is evaluated by the nomogram to predict severe myelosuppression and receives clinical intervention, leading to more net benefits than the intervention-all scheme and intervention-none scheme if the Pt of the patient is approximately between 15 and 60%.

To validate the sensitivity and specificity, we employed the AUC of ROC to evaluate the predictive nomogram model and we found that the AUC values of this nomogram were 0.725 and 0.727 in the training and validation groups, respectively (Fig. 2). The calibration curves indicated that the nomogram yielded acceptable agreement between the nomogram-predicted values and actual values for severe myelosuppression in training and validation groups of SCLC patients following the first-line chemotherapy (Fig. 3). The DCA displayed that the nomogram prediction of severe myelosuppression in SCLC patients following the first-line chemotherapy added more net benefits than the treat-all scheme and treat-none scheme if the range of threshold probability (Pt) was between 15 and 60% in the training and validation groups (Fig. 4). Hence, the nomogram had good discrimination, acceptable calibration, and fine clinical usefulness in predicting the occurrence of severe myelosuppression in SCLC patients following the first-line chemotherapy.

## Discussion

SCLC is characterized by high malignancy, high mortality rate, and limited treatment options, with few alternatives for the three decades^1–4^. Currently, the standard care relies on highly myelotoxic chemotherapy (mainly EP and EC regimens), and many elderly patients with multiple comorbid conditions are prone to develop chemotherapy-induced myelosuppression^16^. Therefore, it is critical to identify the risk factors to predict the occurrence of severe myelosuppression in SCLC patients following the first-line chemotherapy. In this study, we used univariate and multivariate analyses of potential prognostic factors for severe myelosuppression in a group of SCLC patients, according to their demographic and clinical parameters. Age and tumor staging were extracted as predictors to establish the nomogram to predict severe myelosuppression in SCLC patients following the first-line chemotherapy. Age was one of the risk factors for myelosuppression caused by chemotherapy in this study, similar to previous studies^17–19^. The susceptibility of elderly patients to chemotherapy-related severe myelosuppression may stem from a reduction in pluripotent hematopoietic stem cells (PHSC) and their responses to hematopoietic cytokines as well as inhibition of hemopoiesis by inflammatory cytokines, such as interleukin 6 (IL-6) and tumor necrosis factor (TNF)^20,21^. Furthermore, it was not surprising to see that tumor staging (ES type) was a risk factor of severe myelosuppression in SCLC patients following the first-line chemotherapy because the ES type of SCLC usually had distant metastasis and the tumor extended to the bone, liver, brain and malignant pleural effusion as well as other organs. Patients with ES type of SCLC usually have worse physical function and status and are more prone to develop severe myelosuppression following the chemotherapy. Because of its comprehensive effects, we extracted the tumor staging as a predictor for the nomogram model. Other related studies have suggested that the risk factors for chemotherapy-induced myelosuppression include low albumin, history of chemotherapy, low BMI and performance status (PS), gender, etc.^17,19^, which are not consistent with our study. In this study, PS (equivalent to KPS) was significant in univariate analysis, but not in multivariate regression analysis. Other factors in either univariate analysis or multivariate logistic regression analysis did not show any significance in predicting severe myelosuppression induced by first-line chemotherapy in SCLC patients, so they have not been used as predictors to establish the nomogram. This may be mainly related to different chemotherapy regimens. In addition, the criteria of low albumin in these studies are different.

We validated this nomogram in the training and validation groups of SCLC patients. We found that the AUC values were 0.725 and 0.727 in the training and validation groups, respectively, and the nomogram yielded acceptable calibration curves in the two groups. Hence, the nomogram could generate a specific probability for severe myelosuppression using the predictive variables available before each chemotherapy after we calculated the total points from each variable of an individual patient. For example, an 80-year-old male SCLC patient with distant metastasis to the brain and liver can be calculated by the nomogram with Age, 80 years old (100 points), tumor staging, and ES (74 points), resulting in the sum of 174 points, which leads to a probability of 0.54 (54%) for the occurrence of severe myelosuppression. Therefore, both physicians and patients can predict the risk of severe myelosuppression using this intuitive and easy-to-use scoring system and rationalize the clinical decision on any adjuvant therapies before or after chemotherapy, such as prophylaxis of colony-stimulating factor (CSF) or others. Hence, this nomogram may be clinically valuable for the prediction and prevention of severe myelosuppression in SCLC patients following the first-line chemotherapy.

It remains challengeable to decide whether additional treatments or cares are necessary for individual patients during the clinical practices^22^. The DCA can assess the utility of models for decision making by plotting net benefit at a range of clinically reasonable thresholds probability (Pt)^23^. Pt is located at a level, where the expected benefit of the clinical intervention is equal to the expected benefit of avoiding the clinical intervention. It depends on the complexity of the clinical intervention and the willingness of both doctor and patient to the intervention. If the intervention for severe myelosuppression is effective and has fewer side effects, and the patient is eager to treat, the Pt value is smaller. We evaluated the value in decision making using the DCA and found that the nomogram for predicting severe myelosuppression in SCLC patients following the first-line chemotherapy was more beneficial than the intervention-all scheme and intervention -none scheme when the Pt was between 15 and 60%. For example, if a physician’s or a patient’s Pt is 30%, the probability of severe myelosuppression predicted by this nomogram is 31%-60%. After chemotherapy or even before chemotherapy, doctors will provide CSF or other drugs in time to prevent severe myelosuppression, instead of waiting for severe myelosuppression before the intervention. Thus, this predictive nomogram can be beneficial in making the clinical decision to prevent chemotherapy-induced severe myelosuppression in SCLC patients, particularly in the patients with Pt of 15–60%, to ensure that chemotherapy could be implemented completely, and to improve the remission rate and cure rate of SCLC patients.

We recognized that this study had several limitations. First, this was a retrospective investigation with an unavoidable selective bias although we used the same inclusion and exclusion criteria in the validation and training groups. Second, all patients were from a single medical center, and the nomogram model was not validated in an external cohort although the data in the validation and training cohorts came from different periods. Third, because the clinical application of genetic testing for SCLC patients is not conventional, our study did not include any genetic markers, which are associated with severe myelosuppression in tumor patients^18,24,25^. Fourth, a small number of patients in this study began to intervene at the 2 degrees of myelosuppression to avoid the occurrence of severe myelosuppression. Although there was a certain bias, it can better reflect the rules of data in the real-world study. Therefore, it is necessary to conduct further prospective studies to screen more novel and practical factors and verify the nomogram in multiple centers.

In conclusion, we established and validated a nomogram for predicting severe myelosuppression in SCLC patients following the first-line chemotherapy. The nomogram in our study objectively and accurately predict the probability and it is clinically valuable for the prediction and prevention of severe myelosuppression in SCLC patients.

## Data Availability

The data that support the findings of this study are available from the corresponding author upon reasonable request.

## References

[1] Amini A, Byers LA, Welsh JW, Komaki RU (2014). Progress in the management of limited stage small cell lung cancer. Cancer.

[2] Oronsky B, Reid TR, Oronsky A, Carter CA (2017). What’s new in SCLC? A review. Neoplasia.

[3] Wang S (2017). Survival changes in patients with small cell lung cancer and disparities between different sexes, socioeconomic statuses and ages. Sci. Rep..

[4] Schmid S, Fruh M (2018). Immune checkpoint inhibitors and small cell lung cancer: What’s new?. J. Thorac. Dis..

[5] Xie D (2015). Nomograms predict overall survival for patients with small-cell lung cancer incorporating pretreatment peripheral blood markers. J. Thorac. Oncol..

[6] Paz-Ares L (2019). Durvalumab plus platinum-etoposide versus platinum-etoposide in first-line treatment of extensive-stage small-cell lung cancer (CASPIAN): A randomised, controlled, open-label, phase 3 trial. Lancet (London, England).

[7] Evans WK (1985). VP-16 and cisplatin as first-line therapy for small-cell lung cancer. J. Clin. Oncol..

[8] Pietanza MC, Byers LA, Minna JD, Rudin CM (2015). Small cell lung cancer: Will recent progress lead to improved outcomes?. Clin. Cancer Res..

[9] Horn L (2018). First-line Atezolizumab plus chemotherapy in extensive-stage small-cell lung cancer. N. Engl. J. Med..

[10] Pacheco J, Bunn PA (2019). Advancements in small-cell lung cancer: The changing landscape following IMpower-133. Clin. Lung Cancer.

[11] Jones GS, Elimian K, Baldwin DR, Hubbard RB, McKeever TM (2020). A systematic review of survival following anti-cancer treatment for small cell lung cancer. Lung Cancer.

[12] Balachandran VP, Gonen M, Smith JJ, DeMatteo RP (2015). Nomograms in oncology: More than meets the eye. Lancet Oncol..

[13] Consultation WHOE (2004). Appropriate body-mass index for Asian populations and its implications for policy and intervention strategies. Lancet (London, England).

[14] Collins GS, Reitsma JB, Altman DG, Moons KG (2015). Transparent reporting of a multivariable prediction model for individual prognosis or diagnosis (TRIPOD): The TRIPOD statement. Ann. Intern. Med..

[15] Vickers AJ, Elkin EB (2006). Decision curve analysis: A novel method for evaluating prediction models. Med. Decis. Mak..

[16] Weiss JM (2019). Myelopreservation with the CDK4/6 inhibitor trilaciclib in patients with small-cell lung cancer receiving first-line chemotherapy: A phase Ib/randomized phase II trial. Ann. Oncol..

[17] Pettengell R (2009). Multivariate analysis of febrile neutropenia occurrence in patients with non-Hodgkin lymphoma: Data from the INC-EU prospective observational european neutropenia study. Br. J. Haematol..

[18] Cao S (2016). Genome-wide association study of myelosuppression in non-small-cell lung cancer patients with platinum-based chemotherapy. Pharmacogenom. J..

[19] Jiang N, Chen XC, Zhao Y (2013). Analysis of the risk factors for myelosuppression after concurrent chemoradiotherapy for patients with advanced non-small cell lung cancer. Support Care Cancer.

[20] Balducci L (2003). Myelosuppression and its consequences in elderly patients with cancer. Oncology.

[21] Balducci L, Hardy CL, Lyman GH (2000). Hemopoietic reserve in the older cancer patient: Clinical and economic considerations. Cancer Control J. Moffitt Cancer Center.

[22] Huang YQ (2016). Development and validation of a radiomics nomogram for preoperative prediction of lymph node metastasis in colorectal cancer. J. Clin. Oncol..

[23] Van Calster B (2018). Reporting and interpreting decision curve analysis: A guide for investigators. Eur. Urol..

[24] Yang Z, Liu Z (2019). Potentially functional variants of autophagy-related genes are associated with the efficacy and toxicity of radiotherapy in patients with nasopharyngeal carcinoma. Mol. Genet. Genom. Med..

[25] Björn N (2020). Whole-genome sequencing and gene network modules predict gemcitabine/carboplatin-induced myelosuppression in non-small cell lung cancer patients. NPJ Syst. Biol. Appl..

[26] Fitzgerald M, Saville BR, Lewis RJ (2015). Decision curve analysis. JAMA.

